# Evolution not Revolution: Nutrition and Obesity

**DOI:** 10.3390/nu9050519

**Published:** 2017-05-20

**Authors:** Elaine C. Rush, Mary R. Yan

**Affiliations:** 1 AUT Food Network, Auckland University of Technology, Auckland 1142, New Zealand; maryan@aut.ac.nz; 2 Community and Social Sciences, Unitec Institute of Technology, Auckland 1025, New Zealand

**Keywords:** nutrition, social influence, food choice, obesity, sustainable nutrition, agriculture, food industry

## Abstract

The increasing prevalence of obesity over the course of life is a global health challenge because of its strong and positive association with significant health problems such as type 2 diabetes, cardiovascular disease, stroke, and some cancers. The complex causes and drivers of obesity include genetic factors, social, ecological and political influences, food production and supply, and dietary patterns. Public health messages and government food and activity guidelines have little impact; the retail food environment has many low-priced, nutrient-poor, but energy-dense products and there is a gap between what an individual knows and what they do. Public health and education services need legislation to mandate supportive environments and promote food literacy. Two New Zealand case studies of proof-of-principle of positive change are described: Project Energize and Under 5 Energize as exemplars of school environment change, and the development of the Nothing Else™ healthier snack bar as an example of working with the food industry. Changes in food literacy alongside food supply will contribute in the long term to positive effects on the future prevalence of obesity and the onset of non-communicable disease. More cross-disciplinary translational research to inform how to improve the food supply and food literacy will improve the health and wellbeing of the economy and the population.

## 1. Introduction

Good nutrition is a widely-understood premise for good health, but this has not extended to the understanding of the importance of the health of the global food supply and the planet to ensure that sufficient, wholesome food is availably equitably for all inhabitants [1].

A marker of poor nutrition is obesity. Globally obesity is a public health challenge because of its strong and positive association with significant health problems such as type 2 diabetes mellitus (T2DM), cardiovascular disease (CVD), stroke, and some cancers: all non-communicable diseases (NCDs) [2]. In New Zealand, almost one in three (32%) adults are obese [3]. The figure rises to 67% of Pacific Island adults, and 47% of Maori adults and 21% of those living in the most deprived areas. Further, in 2015, one third of New Zealand children were either overweight or obese. The drivers of obesity are complex [4]; they exert the most influence in the early stages of growth and development, and continued exposure over the life course is associated with an increased incidence of obesity and associated NCDs through genetic, environmental and dietary exposure [5]. Public health advice that urges people to eat combinations of wholesome foods, not too much and mainly plants in order to meet the nutrition needs for optimal human and planetary health has been recognized for decades [6,7]. Population dietary recommendations and guidelines are a vital part of strategies to reduce obesity [7]. It is an ongoing scientific and policy-level challenge to provide evidence-based food and nutrition guidelines for public health [8,9]. It is agreed that whole foods should be included in dietary recommendations, in addition to an intake of more fruit and vegetables, whole pulses, nuts and seeds, and less refined and highly processed foods rather than a nutrient-centred focus on sugar, fat and salt.

A health-promoting dietary pattern is not about a single nutrient, food, food group or combination of foods [6]. There are many combinations of foods which, together with not smoking, regular physical activity, supportive social networks, and stress without distress, promote good health. Over many years, the foods we now grow, produce, and process reflect consumer demand, and the food industry has thus responded and created products that are palatable and cheap. The addition of sugar from high yielding sugar cane, maize, and beet to many foods is an example of a change in food formulation that has had adverse consequences on dental health and weight status [10,11]. A person’s nutrition status is largely dependent on the quality and quantity of foods eaten, as well as the lifestyle which has environmental, social ecological, and political influences on self-determining adults’ personal knowledge and beliefs [12,13]. Furthermore, there are lifestyle and intergenerational implications for the arguably improved nutrition of mothers before pregnancy, children in the first 1000 days of life, and in adolescence; all times of rapid growth that may reduce the risk of NCDs [14]. The improvement of public health and population nutrition is currently the focus of many global initiatives of international bodies and governments in order to reduce obesity [4,15] and promote sustainable agriculture [16]. This commentary aims to overview essential relationships and the interdependence of food systems, as well as environmental and social ecology, and to look at potential strategies and examples where improvements in the food supply and population nutrition could be effected.

## 2. Food Production and Nutrition

### 2.1. Nutrients in Foods and Farming—Quantity versus Quality

About ten thousand years ago, planting crops emerged as a primary source of food for humans, and today cereal crops contribute around 50% of calories to the global diet [17]. Farmers and the agricultural industry know that to grow healthy plants, we need water, healthy soil, sun/warmth, and healthy seeds, and it takes time and planning. In turn, these plants feed animals including humans. In farming, nutritio—nally depleted soil and lack of water may contribute to the loss of nutrients [18] further down the food chain. In order to feed the rapidly growing population, the use of synthetic fertilizers, pesticides, and genetic modification to increase productivity have also contributed to nutrient depletion [19]. It is common that fruits and vegetables are picked early for a longer period of storage, but this is associated with the loss of nutrients. For example, brassicas will have up to 50% less vitamin C if they are stored for long periods of time [20].

Particularly for small island nations such as Kiribati, global warming and the associated rise in the sea level reduces the area of land suitable for cropping. Adverse weather events and natural disasters also impact the food supply and appear to be increasing in frequency. In addition, urbanization is associated with the loss of market gardens; town planners recognize the need for a local source of fruit and vegetables, yet heavily rely on imported and highly processed foods.

The challenge of food distribution, particularly with increased urbanization and global trade agreements, means that there are compromises made. New Zealand, with a population of 4.8 million, produces enough food to feed more than 40 million people (personal communication, Ian Ferguson) but the nutritional status and food security of New Zealand’s people are not optimal [21]. In 2011, the prevalence of obesity was 30%, only two thirds of adults reported eating the recommended three or more servings of vegetables each day, and less (60%) reported eating the recommended two or more servings of fruit each day, while only 60% of households reported that they were fully or almost food secure all of the time.

### 2.2. Food Processing

From “farm to fork” food resources are processed to increase food safety, to enhance food texture, color, taste, and flavor, and to reduce cost [22]. With the beneficial impact initiated by global industrialization, highly processed food might lose much of its nutrition value such as protein and fiber so that is can be stored longer. For example, when whole grains are refined, the bran and germ are removed from the endosperm. Furthermore, highly processed foods are often energy-dense and high in refined sugar, salt, and saturated fat [23,24,25]. Sugars are commonly added to accommodate human taste preference, even in the early farming stage. High fat and sugar content and deep frying of foods including takeaway foods lead to the overconsumption of foods and are positively related to body weight gain and obesity due to their highly palatable [26] and weak satiation properties [27]. High salt intake is associated with elevated blood pressure and obesity [28].

Processed and packaged foods often contain additives and preservatives to make food more appealing and to enhance taste. However, additives and preservatives are often associated with foods with reduced nutrient density and may be associated with both perceived [29] and real health problems [30].

## 3. Environmental and Social Ecological Influences

### 3.1. Food Market Globalization

During the last three decades, the human social environment has become a key contributor to the prevalence of the obesity epidemic [31,32]. The growth of diet-related chronic diseases has been related to the rapid change in lifestyle, driven by the increasing rate of industrialization, urbanization, and market globalization [33]. Even though economic development has improved the nutrition and healthcare conditions of populations, it has also led to the rapid increase in the prevalence of obesity, diabetes and heart disease. The increased availability of food, especially energy-dense foods (e.g., high intakes of animal source foods, dairy, and sugar-enhanced foods), and low energy expenditure have significant negative consequences and cause the predisposition of people to obesity [34].

### 3.2. Plethora of Fast Food, Convenient, and Highly Processed Foods and Soft Drinks

Due to the changes in the human social environment and the rise in disposable income, changes in food supply and demand have altered dietary patterns. Fast foods, high in fat and salt, are readily available in numerous food outlets. The booming of fast foods at a lower cost, convenience foods, and 24-hour shopping for some means that preparing meals at home is unnecessary. Fast food consumption is highly related to a higher energy intake and poor nutrition. Eating fast food more than twice a week is associated with an increased risk of weight gain to the point of obesity [35]. Excess fast food consumption is related to the increasing prevalence of CVD and T2DM [36], particularly in developing countries [37]. Soft drinks, also called sugary drinks, have become a widely-consumed drink since the early 19th century. Coca Cola and Pepsi are the two of the most popular brands that are sold successfully over the world, accounting for more than one third of all global sales of soft drinks [38]. Sugary drink consumption is positively and globally linked to overweight, obesity and diabetes [39] as well as to tooth decay [40]. 

### 3.3. Food Marketing

Food advertisements and marketing strategies have a huge investment by the food industry and an impact on people’s food choices, in particular those of children. Contemporaneous changes in technology and lifestyles mean children and youth spend more time in front of television and computer screens. Television advertisements and sales promotions undoubtedly increase purchase intent and consumption [41]. Children’s exposure to food advertisements and marketing of energy-dense, high sugar, high fat food products such as fast foods and sweetened drinks is associated with risk for obesity [42], and means that early on children need to develop a better understanding of what foods are better for them and why.

### 3.4. Parental Modeling

Family is a basic and fundamental unit in society, and therefore generally a key influence factor in children’s eating behaviors. Parents, in particular mothers, determine what to buy, how much to store at home, and how to prepre food [43]. The availability of healthier foods such as a range of fruits and vegatables in the home is positively associated with healthier eating patterns [43]. 

## 4. Potential Strategies to Improve Nutrition and Reduce Risk for Non-Communicable Diseases 

### 4.1. Food Literacy

Although family environment is the proximal and key influence on children’s eating habits, with modern lifestyles there is much less time spent on food preparation and more convenience foods purchased. It is recognized that food literacy, where individuals and communities are ‘equipped’ with knowledge and skills that enable them to make healthier food choices and to prepare food, is essential for better choices and sustained behavior change to be possible [44].

Many randomized controlled interventions to prevent or reduce obesity and promote healthy weight gain have been undertaken in school and workplace environments [12], where children and adults spend a considerable amount of time. Therefore, schools and workplaces are suitable settings for the implementation of food and nutrition policies through changes in the environment and modelled practice and leadership of teachers and managers.

Communication about healthy eating needs to be more than telling; it should be about showing how and be associated with environmental change to support the behavior, for example, replacing less heathy food products with healthier alternatives such as the provision of chilled and filtered water and fruit in schools. Interventions in school or work will and have shown an improvement on eating behaviors which translates into public health gains.

Public health messages and government food and activity guidelines are often not followed, and the food environment does not support healthy eating. There is a gap between what is known and what happens. There is a need for public health services and legislation to provide supportive environments, such as in schools for children, and to promote food literacy by showing populations how to eat better.

### 4.2. The Energize Way

Energize is a New Zealand exemplar of a regional programme showing how to put knowledge into practice, and improve the food and physical activity environment in addition to the food and activity literacy of teachers and children. Favorable outcomes in a 2005 to 2006 randomized controlled trial in 124 primary (year 1 to 8) schools led to a roll-out of Energize to all (242) schools in the Waikato region and 70 schools in other regions, including 53,000 children [45]. The Waikato team celebrated 10 years of progress in 2015. Ongoing evaluation and development of Project Energize has shown it to be sustainable, associated with a lower prevalence of obesity and higher physical fitness [46], as well as cost-effective from the point of view of health treatment costs attributable to obesity. Over the lifecourse one quality adjusted life year (QALY) which equates to a year of life in perfect health would cost between $18,000 and $30,000) and was considered affordable at $45/child/year as a childhood health programme [47]. The programme's unique community-based approach is inclusive of all children in school and reaches a population that comprises 42% of indigenous children, Māori. The nutrition messages of Energize include promoting water and milk as the best drinks, increasing intakes in fruit and vegetables, reducing the availability of high-energy/low-nutrient foods, and encouraging breakfast. All actions are cognizant of the need to keep cost down and recommend available foods. School gardening is supported.

### 4.3. Changes in the Food Supply

Changes in the food supply to make healthy food choices easier have potential effects on public health. The improvement of the food environment involves the food industry working with organizations such as universities and non-governmental agencies, for example, the New Zealand Heart Foundation. Through such collaborations, the reduction of salt is one successful example [48].

Food systems are complex, thus benchmarking and monitoring of effective changes in policy at a global level is underway [49]. At a micro level, there is a need for local food businesses to be supported to make change. As a proof-of-principle case study, the development of the Nothing Else™ healthier snack bar demonstrates one small step towards providing a food environment that is supportive of a healthier diet.

### 4.4. Nothing Else™ Snack Bar

Nothing Else™ started as a label, designed to be printed on the front of a packaged food [50]. The label, in the shape of a circular band, lists all the ingredients in a clear, legible font to provide “up-front” information. A manifesto with the tagline “the Upfront Brand” specifies that ingredients should be natural and familiar with no preservatives, no additives and nothing artificial. The focus is on the consumer and supporting the social practice of sustainable consumption [51].

In partnership with a food manufacturer who baked and packaged the bars, design expertise for the food label from within the university and monitoring of sales through the food outlets at the Auckland University of Technology it has shown that the bar is commercially viable [52]. The Nothing Else™ bar has a favorable effect on acute glycemic response and satiety [53]. In addition, daily consumption of Nothing Else™ bars as a snack replacement for 6 weeks was acceptable and associated with favorable reductions in glycated hemoglobin [54]. Habitual snacking behavior has the potential to be improved through changes in the food supply. In the longer term, the additive effect of many approaches to the improvement of the food supply and food literacy may reduce the impact of poor nutrition on public health.

## 5. Conclusions

It is clear that nutrition has a substantial impact on public health status, and the quality of foods are determined by environmental and social ecological influences. Better understanding of how to modulate food systems and consumer demand could help us to face current health problems such as obesity and T2DM. It is reasonable to look to food or food patterns rather than individual nutrients as the way forward in disease prevention and healthy life promotion. In terms of changes in food supply, working together with the food industry to develop healthier food products could help consumers to make healthier choices. Furthermore, more cross-disciplinary research would inform efforts to improve the food supply and help reverse the epidemic of obesity.
